# Failure to Medically Optimize Before Total Hip Arthroplasty: Which Modifiable Risk Factor Is the Most Dangerous?

**DOI:** 10.1016/j.artd.2021.05.021

**Published:** 2021-07-05

**Authors:** Joseph M. Statz, Susan M. Odum, Nicholas R. Johnson, Jesse E. Otero

**Affiliations:** a South Bend Orthopaedics, South Bend, IN, USA; b OrthoCarolina Research Institute, Charlotte, NC, USA; c Department of Orthopaedic Surgery, Atrium Health Musculoskeletal Institute, Charlotte, NC, USA; d OrthoCarolina Hip and Knee Center, Charlotte, NC, USA

**Keywords:** Hip, Arthroplasty, Total hip arthroplasty, Modifiable risk

## Abstract

**Background:**

There is mounting evidence that smoking, abnormal body mass index (BMI), uncontrolled diabetes, and poor nutritional status are associated with complications after total hip arthroplasty (THA). The goal of the present study was to evaluate the consequences of failure to medically optimize Medicare-eligible patients with respect to these key modifiable health targets by assessing complications in the early postoperative period after THA.

**Methods:**

The National Surgical Quality Improvement Program database was queried for all primary THAs performed in 2018. Data were collected on preoperative serum albumin, BMI, diabetes, and tobacco use as well as postoperative infections, readmissions, complications, and mortality. We identified 47,924 THA patients with a median BMI of 29 kg/m^2^ and age of 72 years, and 60% of whom were female.

**Results:**

We found that preoperative albumin <3.5 g/dL, BMI ≥40 kg/m^2^, tobacco use, and diabetes were all individually associated with increased risk of postoperative complications. Serum albumin <3.5 g/dL was the greatest overall risk factor for infection (odds ratio [OR]: 3.1, 95% confidence interval [CI]: 2.3-4.4, *P* < .0001), readmission (OR: 2.2, 95% CI: 1.9-2.5, *P* < .0001), any complication (OR: 4.2, 95% CI: 3.8-4.6, *P* < .0001), and mortality (OR: 7.5, 95% CI: 5.3-10.6, *P* < .0001).

**Conclusions:**

Low albumin, elevated BMI, tobacco use, and diabetes are associated with increased risk of postoperative infection, readmission, any complication, and mortality after primary THA. Low albumin poses the greatest risk of these. Preoperative optimization should be obtained in all patients before elective surgery, and the final decision for surgery should be individually made between a surgeon and patient.

**Level of Evidence:**

IV.

## Introduction

Primary total hip arthroplasty (THA) is one of the most successful surgeries in the world today and is associated with extremely high postoperative patient satisfaction rates and functional scores with a low complication rate. However, when complications do occur, they can be devastating for the patient and surgeon alike. The most common complications seen after THA include infection, periprosthetic fracture, instability, aseptic loosening, transfusion, and deep venous thrombosis [[1], [2], [3], [4]].

Recently, there has been a movement toward ensuring patients are preoperatively medically optimized before undergoing THA [[10], [11], [12], [13], [14], [15], [5], [6], [7], [8], [9]]. This movement is based on increasing evidence that patients with complex medical problems and/or modifiable behaviors are at higher risk of complications after THA [11,[16], [17], [18], [19], [20], [21], [22], [23], [24], [25]]. Although not all risk factors are modifiable, certain ones are. There is mounting evidence that smoking, [[1], [2], [3], [4]] abnormal body mass index (BMI), [11,[26], [27], [28], [29], [30], [31], [32], [33], [34], [35], [36], [37], [38], [39], [40], [41], [42], [43]] uncontrolled diabetes, [[44], [45], [46], [47], [48], [49], [50], [51], [52], [53]] and poor nutritional status [[54], [55], [56], [57]] are associated with poorer THA outcomes. There are also some data to suggest that improving these risk factors is possible before THA and decreases complications [[57], [58], [59], [60], [61], [62], [63], [64], [65], [66], [67]].

Bundled Payments for Care Improvement was implemented by Centers for Medicare & Medicaid Services almost a decade ago and has been shown to reduce complications and cost after total joint arthroplasty [68]. Alternative episode of care initiatives have also been introduced with the goal of reducing complications and cost through preoperative patient health optimization [69]. The goal of the present study was to evaluate the consequences of failure to optimize Medicare-eligible patients in key modifiable health targets by assessing complications in the early postoperative period after THA. We hypothesized that a greater burden of nonoptimized targets would result in a higher complication rate. Furthermore, we believed that preoperative optimization of these risk factors would decrease the risk of complications compared with patients who did not undergo preoperative optimization.

## Material and methods

The data for this study were sourced from the 2017 and 2018 Participant Use Data Files (PUF) from the American College of Surgeons National Surgical Quality Improvement Program (NSQIP) database. The PUFs are patient-level, aggregate data that are compliant with the Health Insurance Portability and Accountability Act. As such, PUFs do not contain any patient or hospital identifiers, and with the execution of a data use agreement, any research with these data do not require the review and approval of an institutional review board. All primary THA surgeries between January 2017 and December 2018 were identified using the 27,130 Current Procedural Terminology code, and data on patient medical problems, BMIs, laboratory values, and complications were extracted and analyzed.

### Outcome variables and risk factors

We extracted patient data on sex, age, BMI, serum albumin levels, current tobacco use, and diagnosis of diabetes along with insulin dependence. We additionally recorded the occurrence of infection, readmission, any complication, and mortality within 90 days postoperatively. The category “any complication” encompassed the following complications: superficial and deep wound infection, organ space infection, wound dehiscence, pneumonia, unplanned intubation, deep venous thrombosis, pulmonary embolism, renal insufficiency, stroke, coma, cardiac arrest, myocardial infarction, postoperative transfusion, implant failure, sepsis, septic shock, and mortality.

### Patient demographics

We identified a total of 85,880 THAs. After excluding 37,956 patients younger than 65 years, a total of 47,924 THAs were included for analysis. The median BMI was 28.7 (interquartile range: 25.2-32.8 kg/m^2^), age was 72 (interquartile range: 68-78) years, and 60.2% were female.

### Statistical methods

BMI, albumin, tobacco use, and diabetes were analyzed as dichotomous variables. BMI <40 kg/m^2^ was compared to BMI ≥40 kg/m, albumin <3.5 g/dL was compared to ≥3.5 g/dL, current use of tobacco was compared to no current use, and having insulin-dependent diabetes was compared to having non–insulin-dependent diabetes as well as to not having diabetes. If a patient was missing values for a risk factor, they were excluded in the analysis of that specific risk factor. We also compared patients who were medically optimized in risk factors of BMI, albumin, smoking, and diabetes to those not optimized in one or more risk factors. If patients had missing data for one or more of these risk factors, they were considered not medically optimized if they had any risk factor that was not optimized. If they had missing data but were optimized in all the risk factors for which data were present, they were excluded from analysis of patients who were medically optimized vs those not medically optimized [2].

At the bivariate level, categorical variables were statistically compared using Chi-squared tests. For all categorical data, frequency and percentages are reported. Four multivariable logistic regression models were used to evaluate the risk factors associated with infection, readmission, any complication, and mortality. Model testing revealed multicollinearity when the optimized variable was included with each individual risk factor. Furthermore, model fit statistics indicated that the regression models that included BMI, albumin, tobacco use, and diabetes were the best fitting models. For all logistic regression models, odds ratios (ORs) and Wald 95% confidence intervals (CIs) are reported. An apriori level of significance of 0.05 was defined. All data were managed and analyzed using SAS/STAT software version 9.4 (SAS Institute Inc., Cary, NC).

### Sources of funding

No external sources of funding were used for this study.

## Results

### Risk factor optimization

Of the 47,924 THAs in this study, 28,468 (59.4%) had data available to determine preoperative medical optimization. Of these, 35,251 (70.3%) were considered optimized with BMI <40 kg/m^2^, albumin ≥3.5 g/dL, no current tobacco use, and no diabetes. Looking at each risk factor individually, BMI data were available for 47,541 (99.2%) THAs, albumin data were available for 25,641 (53.5%) THAs, and tobacco use and diabetes data were available for 47,924 (100%) THAs. Of the THAs with data available, there were 2292 (4.8%) THAs with BMI ≥40 kg/m^2^, 2242 (8.7%) THAs with albumin <3.5 g/dL, 3177 (6.6%) THAs who were current tobacco users, 1628 (3.4%) THAs with insulin-dependent diabetes, and 5187 (10.8%) THAs with non–insulin-dependent diabetes.

### Risk factors for infection

Infection was more common in the presence of any of the studied risk factors. The risk factor that was most highly correlated with postoperative infection was BMI ≥40 kg/m^2^ (OR: 3.196, 95% CI: 2.32-4.404, *P* < .0001), followed by albumin <3.5 g/dL (OR: 3.147, 95% CI: 2.419-4.093, *P* < .0001), diabetes (OR: 1.392, 95% CI: 1.065-1.818, *P* = .0011), and tobacco use (OR: 1.267, 95% CI: 0.858-1.871, *P* = .2727). Patients who were not optimized preoperatively were more likely to develop an infection postoperatively as compared to patients who were optimized preoperatively (2.5% vs 0.9%, respectively, *P* < .0001). Infection risk data are summarized in Table 1.

**Table 1:** Risk factors for postoperative infection.

Demographic variable	Infection, n (%)	No infection, n (%)	OR (95% CI)	*P* value
Albumin	336 (1.3)	25,305 (98.7)		
<3.5 g/dL	81 (3.6)	2161 (96.4)	3.147 (2.419-4.093)	*P* < .0001
≥3.5 g/dL	255 (1.1)	23144 (98.9)		
BMI	637 (1.3)	46904 (98.7)		
≥40 kg/m^2^	92 (4.0)	2200 (96.0)	3.196 (2.32-4.404)	*P* < .0001
<40 kg/m^2^	545 (1.2)	44704 (98.8)		
Tobacco use	650 (1.4)	47274 (98.6)		
Yes	50 (1.6)	3127 (98.4)	1.267 (0.858-1.871)	*P* = .2727
No	600 (1.3)	44147 (98.7)		
Diabetes	650 (1.4)	47274 (98.6)		
Insulin-dependent	31 (1.9)	1597 (98.1)	1.392 (1.065-1.818)	*P* = .0011
Non–insulin-dependent	94 (1.8)	5093 (98.2)		
No	525 (1.3)	40584 (97.7)		
Optimized	399 (1.4)	28069 (98.6)		
No	211 (2.5)	8233(97.5)	(-)	*P* < .0001
Yes	188 (0.9)	19836 (99.1)		

### Risk factors for readmission

Readmission was more common in the presence of any of the studied risk factors. The risk factor that was most highly correlated with postoperative readmission was albumin <3.5 g/dL (OR: 2.173, 95% CI: 1.865-2.532, *P* < .0001), followed by BMI ≥40 kg/m^2^ (OR: 1.79, 95% CI: 1.439-2.226, *P* < .0001), tobacco use (OR: 1.389, 95% CI: 1.13-1.706, *P* = .0003), and diabetes (OR: 1.225, 95% CI: 1.057-1.419, *P* < .0001). Patients that were not optimized preoperatively were more likely to be readmitted postoperatively than patients who were optimized preoperatively (7.0% vs 4.3%, respectively, *P* < .0001). Readmission risk data are summarized in Table 2.

**Table 2:** Risk factors for postoperative readmission.

Demographic variable	Readmission, n (%)	No readmission, n (%)	OR (95% CI)	*P* value
Albumin	1282 (5.0)	24359 (95.0)		
<3.5 g/dL	231 (10.3)	2011 (89.7)	2.173 (1.865-2.532)	*P* < .0001
≥3.5 g/dL	1051 (4.5)	22348 (95.5)		
BMI	2134 (4.5)	45407 (95.5)		
≥40 kg/m^2^	168 (7.3)	2124 (92.7)	1.79 (1.439-2.226)	*P* < .0001
<40 kg/m^2^	1966 (4.3)	43283 (95.7)		
Tobacco use	2154 (4.5)	45770 (95.5)		
Yes	184 (5.8)	2993 (94.2)	1.389 (1.13-1.706)	*P* = .0003
No	1970 (4.4)	42777 (95.6)		
Diabetes	2154 (4.5)	45770 (95.5)		
Insulin-dependent	118 (7.2)	1510 (92.8)	1.225 (1.057-1.419)	*P* < .0001
Non–insulin-dependent	258 (5.0)	4929 (95.0)		
No	1778 (4.3)	39331 (95.7)		
Optimized	1444 (5.1)	27024 (94.9)		
No	592 (7.0)	7852 (93.0)	(-)	*P* < .0001
Yes	852 (4.3)	19172 (95.7)		

### Risk factors for complication

Postoperative complications were more common in the presence of any of the studied risk factors. The risk factor that was most highly correlated with any complication was albumin <3.5 g/dL (OR: 4.162, 95% CI: 3.759-4.609, *P* < .0001), followed by diabetes (OR: 1.445, 95% CI: 1.304-1.601, *P* < .0001), tobacco use (OR: 1.192, 95% CI: 1.024-1.387, *P* = .1693), and BMI ≥40 kg/m^2^ (OR: 1.185, 95% CI: 0.994-1.413, *P* = .0003). Patients who were not optimized preoperatively were more likely to develop a complication postoperatively than patients who were optimized preoperatively (16.1% vs 8.8%, respectively, *P* < .0001). Complication risk data are summarized in Table 3.

**Table 3:** Risk factors for postoperative complication.

Demographic variable	Complication, n (%)	No complication, n (%)	OR (95% CI)	*P* value
Albumin	2853 (11.1)	22788 (88.9)		
<3.5 g/dL	706 (31.5)	1536 (68.5)	4.162 (3.759-4.609)	*P* < .001
≥3.5 g/dL	2147 (9.2)	21252 (90.8)		
BMI	4634 (9.7)	42907 (90.3)		
≥40 kg/m^2^	274 (11.9)	2018 (88.1)	1.185 (0.994-1.413)	*P* = .0003
<40 kg/m^2^	4360 (9.6)	40889 (90.4)		
Tobacco use	4717 (9.8)	43207 (90.2)		
Yes	335 (10.5)	2842 (89.5)	1.192 (1.024-1.387)	*P* = .1693
No	4382 (9.8)	40365 (90.2)		
Diabetes	4717 (9.8)	43207 (90.2)		
Insulin-dependent	277 (17.0)	1351 (83.0)	1.445 (1.304-1.601)	*P* < .0001
Non–insulin-dependent	641 (12.4)	4546 (87.6)		
No	3799 (9.2)	37310 (90.8)		
Optimized	3118 (11.0)	25350 (89.0)		
No	1359 (16.1)	7085 (83.9)	(-)	*P* < .0001
Yes	1759 (8.8)	18265 (91.2)		

### Risk factors for mortality

Mortality was more common in the presence of any of the studied risk factors. The risk factor that was most highly correlated with postoperative mortality was albumin <3.5 g/dL (OR: 7.477, 95% CI: 5.251-10.646, *P* < .0001), followed by diabetes (OR: 1.853, 95% CI: 1.251-2.742, *P* < .0001), BMI ≥40 kg/m^2^ (OR: 1.398, 95% CI: 0.672-2.906, *P* = .9183), and tobacco use (OR: 1.216, 95% CI: 0.63-2.348, *P* = .6934). Patients that were not optimized preoperatively were more likely to die postoperatively than patients who were optimized preoperatively (1.0% vs 0.3%, respectively, *P* < .0001). Mortality risk data are summarized in Table 4.

**Table 4:** Risk factors for postoperative mortality.

Demographic variable	Mortality, n (%)	No mortality, n (%)	OR (95% CI)	*P* value
Albumin	137 (0.5)	25504 (99.5)		
<3.5 g/dL	63 (2.8)	2179 (97.2)	7.477 (5.251-10.646)	*P* < .0001
≥3.5 g/dL	74 (0.3)	23325 (99.7)		
BMI	193 (0.4)	47348 (99.6)		
≥40 kg/m^2^	9 (0.4)	2283 (99.6)	1.398 (0.672-2.906)	*P* = .9183
<40 kg/m^2^	184 (0.4)	45065 (99.6)		
Tobacco use	202 (0.4)	47722 (99.6)		
Yes	12 (0.4)	3165 (99.6)	1.216 (0.63-2.348)	*P* = .6934
No	190 (0.4)	44557 (99.6)		
Diabetes	202 (0.4)	47722 (99.6)		
Insulin-dependent	23 (1.4)	1605 (98.6)	1.853 (1.251-2.742)	*P* < .0001
Non–insulin-dependent	26 (0.5)	5161 (99.5)		
No	153 (0.4)	40956 (99.6)		
Optimized	144 (0.5)	28324 (99.5)		
No	84 (1.0)	8360 (99.0)	(-)	*P* < .0001
Yes	60 (0.3)	19964 (99.7)		

## Discussion

In this article, we performed a retrospective review of NSQIP data including all THAs performed in 2018 and sought to determine the increased risk associated with certain preoperative variables and their impact on postoperative outcomes. We found that preoperative albumin <3.5 g/dL, BMI ≥40 kg/m^2^, tobacco use, and diabetes were associated with increased risk of postoperative infection, readmission, any complication, and mortality.

The finding that these risk factors were associated with adverse outcomes is consistent with previous studies [[1], [2], [3], [4]]; [11,[26], [27], [28], [29], [30], [31], [32], [33], [34], [35], [36], [37], [38], [39], [40], [41], [42], [43]]; [[44], [45], [46], [47], [48], [49], [50], [51], [52], [53], [54], [55], [56], [57]]. It is unsurprising that these risk factors would be associated with poorer outcomes after THAs as they are harbingers of impaired wound healing and poor general state of health. The present study lends further evidence that less healthy patients tend to have a higher rate of postoperative complications.

Importantly, we found that preoperative serum albumin <3.5 g/dL was the variable most predictive of these postoperative complications despite being tested for in the smallest number of patients. Serum albumin <3.5 g/dL had odds ratios of 2.317 to 7.221 for the complications examined in this study and a complication rate of up to 17% (Table 3). This finding has led to a change in the senior author’s practice, and now preoperative albumin levels are drawn on every patient undergoing THA. If albumin levels are low, patients are started on supplemental nutritional shakes twice daily and instructed to be in a low-inflammatory diet during their laboratory tests before surgery, and this is continued for at least 6 weeks postoperatively if not indefinitely. Surgery is not delayed if they are otherwise optimized. Patients are instructed to follow up with their primary care physicians within a month postoperatively for long-term management of the malnutrition.

There are many weaknesses of this study. First, its retrospective nature may make it susceptible to selection bias, selecting which patients had preoperative values obtained. However, data are prospectively collected, which mitigates this weakness, and data concerning BMI, tobacco use, and diabetes was available for almost all patients. Second, the presence of diabetes was used in this study as a risk factor, even though this is not modifiable. Once a patient is diagnosed with diabetes, they are considered to have diabetes the rest of their life. It would have been better to use hemoglobin A1C as a risk factor, but this was not available in the NSQIP database. Third, serum albumin levels were only available in 53.5% of the THAs in this study. We recognize that this could be due to a selection bias, which led to sicker patients being more predominantly tested for serum albumin levels. This brings into question the validity of albumin being such an important risk factor for complications. However, the vast majority of THAs for which an albumin level was drawn had values ≥3.5 g/dL, and there were still 25,641 THAs with an associated albumin level, increasing the strength of this association through sheer numbers. Likewise, only 59.4% of THAs had data available to determine preoperative medical optimization, but there were still 28,468 THAs with these data available. Fourth, we were hoping to perform an analysis of additive risks if patients had multiple risk factors. However, there were not enough patients with multiple risk factors to perform such an analysis. For example, there was not a single patient who was “not optimized” in all 4 categories. Fifth, we did not analyze specific complications, reasons for readmission, or reasons for death. However, we aimed to give a broad overview of risk factors and postoperative complications, and getting into such specifics was outside the aims of this study. Sixth, this study attempted to determine risk associate with a failure to optimize patients before THA, but the ability to truly determine this from the NSQIP database could be called into question because of the limitations of the database. This database is only able to give us demographic and laboratory data on patients during the perioperative time. It does not tell us whether any attempt at true preoperative optimization of the aforementioned risk factors was attempted by the patient’s health-care team preoperatively. Thus, it is possible that our data more accurately represent increased risk associated with poor patient selection rather than poor patient optimization. In addition, follow-up is only available short term and is fallible with regard to coding and data entry error.

Strengths of this study include its large numbers, the analysis of multiple risk factors, analysis based on whether a patient was preoperatively optimized or not, and the determination that serum albumin <3.5 g/dL is the most important risk factor for postoperative complications.

Our current practice and recommendation for preoperative optimization is to obtain a thorough history (including tobacco use and history of diabetes), physical examination (including BMI), and x-ray to determine if a patient would benefit from elective primary THA. If the patient’s BMI ≥40 kg/m^2^, if they currently use tobacco, or if the patient has diabetes with a hemoglobin A1C ≥ 8.0%, the patient is informed that his or her risk of surgery is elevated, and weight loss, tobacco cessation, and/or improved diabetes control need to occur before THA to minimize risk. If these factors are optimized, laboratory work is obtained including serum albumin. If the albumin is <3.5 g/dL, surgery is not delayed, but patients are started on supplementation as detailed previously. All THA candidates are sent to a primary care physician for preoperative medical optimization. In addition, we ensure that the patient has other resources available to aid in optimization including a referral to a nutritionist and smoking cessation program.

We do recognize that there is a difference between being medically optimized and meeting all these cutoffs. For example, some patients, due to various medical conditions, may be as optimized as possible with a serum albumin level of <3.5 g/dL. Therefore, we believe it is important to individualize decision-making and to note that the final decision for surgery should be made between an individual surgeon and patient. If nothing else, these data can help guide this individualized decision-making to aid surgeons and patients in making informed decisions for their THAs. Nonetheless, we do believe that preoperative optimization is vital and that it is important to improve modifiable risk factors as much as possible to decrease risk, even if recommended objective cutoffs are not met [[57], [58], [59], [60], [61], [62], [63], [64], [65], [66], [67]].

## Conclusions

Preoperative serum albumin <3.5 g/dL, BMI ≥40 kg/m^2^, tobacco use, and diabetes are associated with increased risk of postoperative infection, readmission, any complication, and mortality after primary THA. Albumin <3.5 g/dL is the most predictive of adverse outcomes. Preoperative optimization should be obtained in all patients before elective surgery, and the final decision for surgery should be individually made between a surgeon and patient.

## Conflicts of interest

The authors declare that they have no known competing financial interests or personal relationships that could have appeared to influence the work reported in this article.

## Appendix A Supplementary data

Conflict of Interest Statement for Statz

Conflict of Interest Statement for Johnson

Conflict of Interest Statement for Odum

Conflict of Interest Statement for Otero
