# Supplementary material for: Failure to Medically Optimize Before Total Hip Arthroplasty: Which Modifiable Risk Factor Is the Most Dangerous?
Source: Arthroplast Today. 2021 Jul 5;10:18–23. doi: 10.1016/j.artd.2021.05.021 (PMC8267488; doi:10.1016/j.artd.2021.05.021)
Supplement: Conflict of Interest Statement for Otero [file mmc4.docx]

# CONFLICT OF INTEREST STATEMENT

***American Association of Hip and Knee Surgeons***

(Adopted from the American Academy of Orthopaedic Surgeons disclosure statement)

The following form **must be filled out completely and submitted by each author (example, 6 authors, 6 forms).**

**All items require a response. If there is no relevant disclosure for a given item, enter "*None*.”**

Manuscript Title **Failure to Medically Optimize Prior to THA:**

**Which Modifiable Risk Factor is the Most Dangerous?**

1. Royalties from a company or supplier (The following conflicts were disclosed)

2. Speakers bureau/paid presentations for a company or supplier (The following conflicts were disclosed)

3A. Paid employee for a company or supplier (The following conflicts were disclosed)

3B. Paid consultant for a company or supplier (The following conflicts were disclosed)

**DePuy, A Johnson & Johnson Company**

3C. Unpaid consultants for a company or supplier (The following conflicts were disclosed)

4. Stock or stock options in a company or supplier (The following conflicts were disclosed)

5. Research support from a company or supplier as a Principal Investigator (The following conflicts were disclosed)

**DePuy, A Johnson & Johnson Company**

6. Other financial or material support from a company or supplier (The following conflicts were disclosed)

7. Royalties, financial or material support from publishers (The following conflicts were disclosed)

8. Medical/Orthopaedic publications editorial/governing board (The following conflicts were disclosed)

9. Board member/committee appointments for a society (The following conflicts were disclosed)

**American Association of Hip and Knee Surgeons**

**Each author must sign AND print or type his/her name, date and submit a separate form**

In addition, one BLINDED Conflict of Interest form (no author names used) should be submitted per manuscript with all author disclosures.


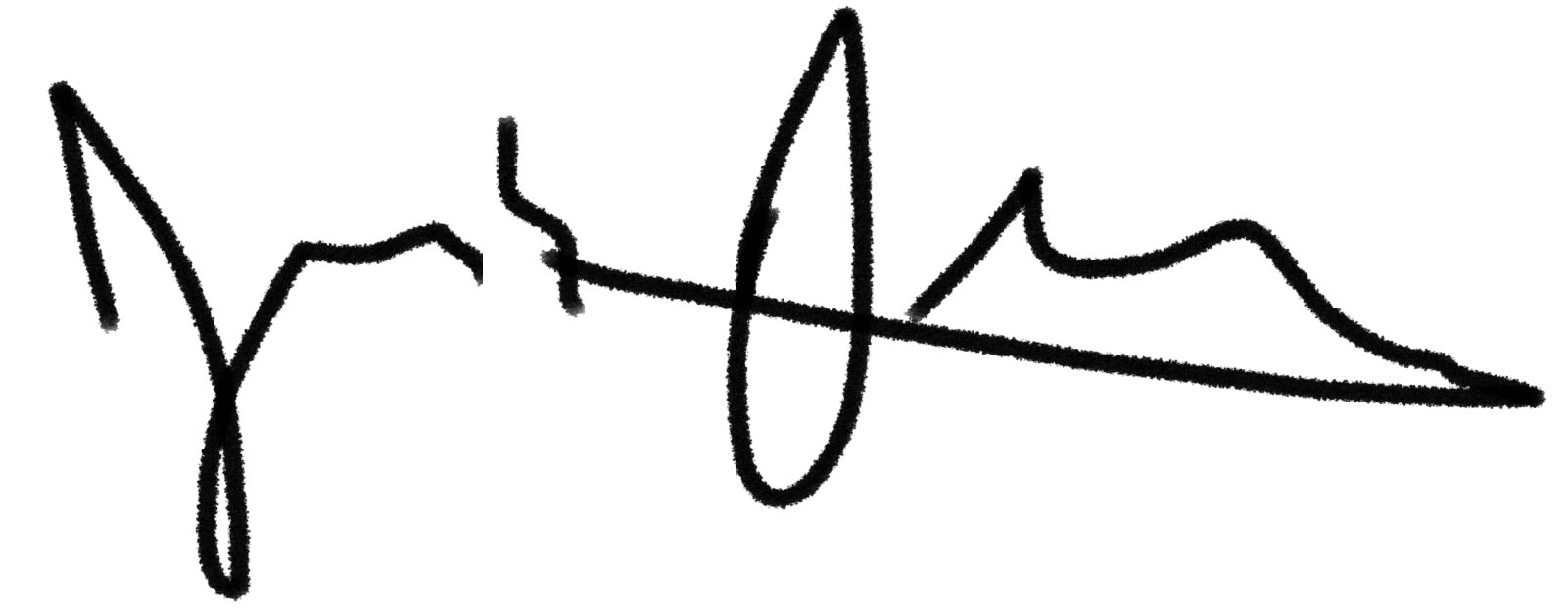


**Jesse E. Otero, M.D., Ph.D.**

01/28/2021

Author Name (Print or Type) Author Signature Date
